# Long-Term Effects of Vineyard Floor Vegetation Management on Grapevine Physiology, Yield and Wine Color Attributes Under Mediterranean Conditions

**DOI:** 10.3390/plants15193046

**Published:** 2026-10-05

**Authors:** Ronit Yaa’ri, Ido Walk, Moshe Sibony, Bat Chen R. Lubin, Jyoti Solanki, Yaakov Goldwasser, Tovit Rosenzweig, Baruch Rubin, Yishai Netzer

**Affiliations:** 1 Chemical Engineering Department, Ariel University, Ariel 4070000, Israel; ronityar@ariel.ac.il (R.Y.); batchenl@ariel.ac.il (B.C.R.L.); jytsolanky269@gmail.com (J.S.); 2 Department of Agriculture and Oenology, Eastern R&D Center, Ariel University, Ariel 4070000, Israel; ido.walk@mail.huji.ac.il; 3 Department of Molecular Biology, Faculty of Natural Sciences, Ariel University, Ariel 40700, Israel; tovitro@ariel.ac.il; 4 RH Smith Institute of Plant Sciences & Genetics in Agriculture, Faculty of Agriculture, Food and Environment, The Hebrew University of Jerusalem, Rehovot 7610001, Israel; moshe.sibony@mail.huji.ac.il (M.S.); yaakov.goldwasser@mail.huji.ac.il (Y.G.)

**Keywords:** cover crops, turf grass, *Festuca arundinacea*, selective herbicide, vine water status, grapevine performance, wine color attribute

## Abstract

Cover crops (CCs) are widely used to improve soil function, support water regulation, and moderate vine vigor. This study evaluated alternative vineyard floor management strategies designed to improve ecosystem services while minimizing excessive competition with grapevines under semiarid Mediterranean conditions. A long-term, three-season field experiment was conducted in Bekoa, Israel, using four treatments: standard-management control, selective herbicide promoting annual winter grasses, interrow tall fescue (*Festuca arundinacea*-TF), and full-TF ground cover. Unlike conventional perennial CCs, the selective herbicide treatment promoted naturally occurring winter grass weeds that completed their life cycle before the dry summer period, thereby limiting prolonged competition with grapevines. Vine water status, vegetative-growth, yield components, and wine-color attributes were monitored. The tall fescue treatments progressively reduced vegetative growth and were associated with more negative stem water potential. Yield declined over the three growing seasons because cluster numbers decreased, with the largest cumulative decline in the tall fescue between-row treatment, followed by the full-cover tall fescue treatment. In contrast, the selective herbicide treatment consistently produced intermediate physiological and agronomic responses. In the third season, wines from the perennial turfgrass treatments exhibited greater color intensity, particularly under full-cover TF, while hue was not significantly affected. Overall, CC-based vineyard floor management affected vine performance and wine color attributes but the response depended on cover-type and duration of use. The selective promotion of naturally occurring winter grasses through targeted herbicide application produced intermediate responses in vine water status, vegetative growth, and yield, suggesting that limiting active ground cover to the wettest part of the season may reduce prolonged summer competition with grapevines.

## 1. Introduction

Vineyard floor management plays a central role in sustainable viticulture, directly affecting soil physical properties, water dynamics, vine vigor, and ultimately, fruit and wine quality [1]. Under Mediterranean conditions, rainfall is concentrated in winter, while summers are hot and dry. As a result, ground-cover strategies must balance their potential benefits to soil health and ecosystem functioning against the risk of excessive competition for water and nutrients [2]. Many Mediterranean vineyards rely on regulated deficit irrigation and canopy management to achieve a desired balance between yield and quality. Therefore, any practice that affects soil water availability or vine vigor can lead to measurable changes in fruit composition and wine attributes [3,4,5,6].

Cover crops (CCs) are defined as non-harvested plant species grown primarily to improve soil and agroecosystem performance. CCs have become an integral part of modern vineyard management and are typically placed between vine rows. Under controlled conditions, CCs are sometimes planted within the rows. Reported benefits include reduced surface runoff and erosion, improved soil structure and organic matter content, reduced weed competition, and enhanced beneficial microbial activity [7]. These benefits are particularly important in sloping or erosion-prone landscapes, where soil loss removes the upper horizon, which is richest in organic matter and nutrients. This soil loss can thus threaten the long-term productivity of a vineyard [8,9]. CCs may also contribute to improved soil quality by increasing organic matter in the soil. As a result, aggregation, infiltration, and overall soil functioning are enhanced [10]. Vineyard systems that maintain CCs often show greater soil organic matter than systems managed with intensive soil disturbance or herbicide-based bare-soil. This pattern has been observed in both seeded CCs and resident vegetation allowed to grow between vine rows [11,12]. Similar responses have also been reported under Mediterranean conditions, including Israeli vineyards. Therefore, maintaining resident winter ground cover can increase the soil organic matter and reduce soil loss compared with spraying or tillage [13].

Additionally, CCs may also help moderate vine vegetative growth, particularly where water availability is high or vine vigor is excessive. This reduction in canopy density can indirectly improve grape composition and air circulation, thereby contributing to lower disease pressure [14,15]. However, during spring and summer, CCs may compete with vines for water and nutrients, especially in Mediterranean climates, potentially leading to yield reductions. The magnitude and direction of these effects depend on the CC species, vine root stock, soil characteristics, and irrigation regime [2,16]. Previous studies have mainly examined permanent CCs or annually sown species removed before summer, while less attention has been given to natural winter vegetation managed to reduce competition with grapevines during the dry season [17]. Under semiarid Mediterranean conditions, limited water availability and the risk of excessive summer competition restrict the adoption of conventional CCs systems [2,18]. These constraints highlight the need for alternative vineyard floor management strategies that retain the benefits of ground cover while minimizing negative impacts on vine performance.

Vineyard-floor management also influences plant biodiversity and vegetation composition. Maintaining spontaneous or less intensively managed ground vegetation generally supports greater biodiversity than intensive tillage or herbicide-based management [11,19]. Over time, repeated mowing, herbicide use, and establishment of perennial vegetation can also alter plant-community succession and functional composition [20,21]. These ecological dynamics are particularly relevant in Mediterranean vineyards, where seasonal vegetation persistence may determine both ecosystem functions and competition with grapevines [2]. Taken together, previous studies indicate that vineyard-floor management involves a trade-off rather than a uniformly beneficial response. Bare-soil or intensive herbicide management minimizes direct competition from ground vegetation but provides fewer soil-cover and biodiversity benefits, whereas permanent vegetation generally improves soil protection and ecological functioning but may increase competition for water and nutrients [11,12,13]. Seasonal or temporary vegetation can provide an intermediate strategy by maintaining ground cover during the wettest part of the year while reducing competition during the dry season. The magnitude of these effects depends on the species used, duration and spatial extent of ground cover, soil properties, climate, and irrigation management [19].

Therefore, the objective of this research was to evaluate vineyard performance under ground-cover strategies adapted to Mediterranean conditions, combining resident winter ground cover, selective herbicide management promoting annual grasses, and seeded perennial turfgrass (*Festuca arundinacea*). We hypothesized that selective promotion of naturally occurring winter grass weeds would provide an intermediate vineyard floor management strategy, retaining many of the agronomic benefits associated with maintaining seasonal vineyard floor vegetation while reducing excessive summer water competition compared with perennial turfgrass systems.

## 2. Results

### 2.1. Leaf Area Index (LAI)

**Figure 1:**
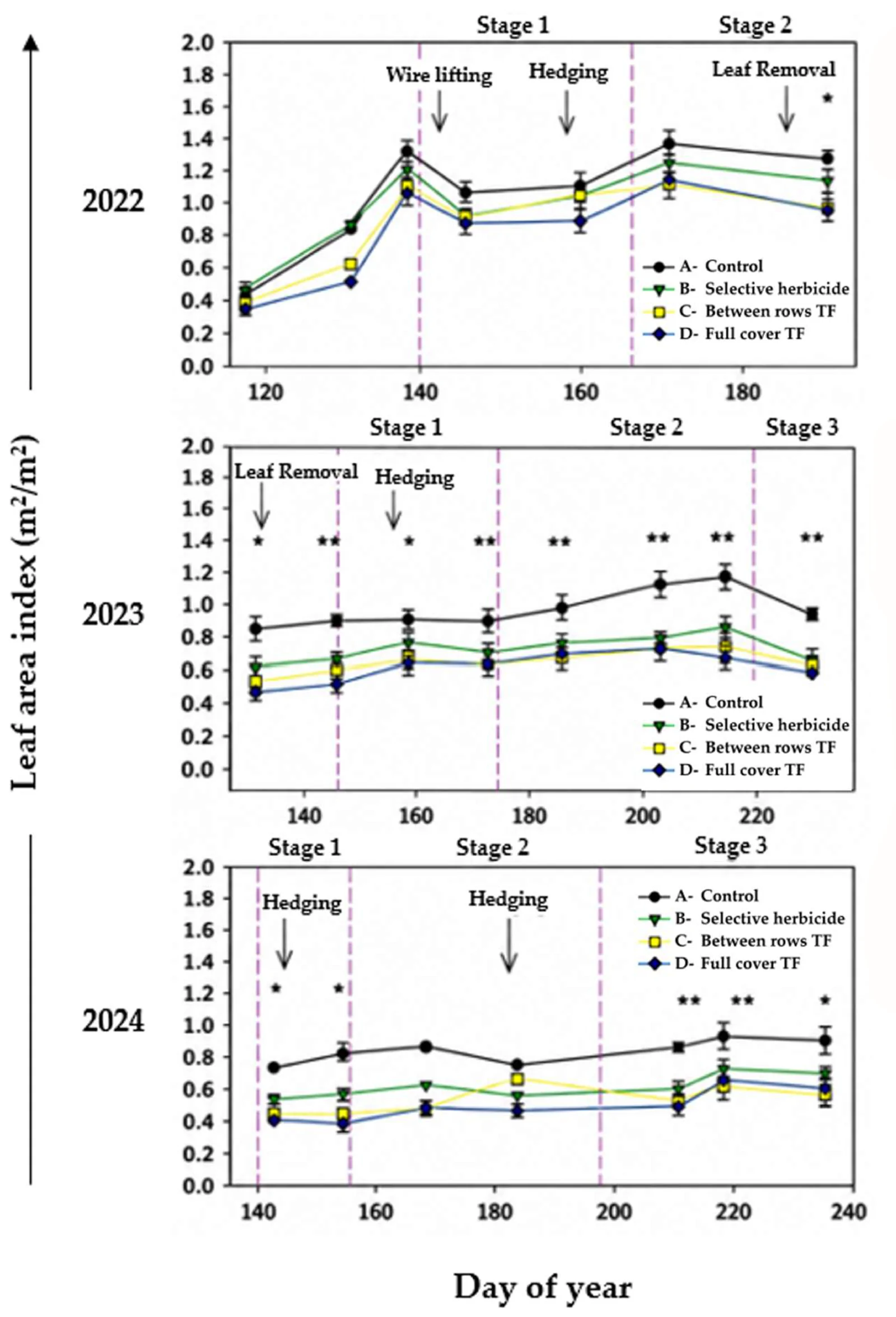
Seasonal dynamics of the LAI under different weed management treatments during year 2022 (**upper**), 2023 (**middle**), and 2024 (**lower**) growing seasons. Treatments included a control (A), a selective herbicide (B), tall fescue between rows (C), and full cover tall fescue (D). The X-axis indicates the day of the year, and the fruit development stages (Stages 1–3) are shown above each graph. The arrows denote canopy management operations. Values are presented as means ± SE (*n* = 5) for 2022 and 2024 (*n* = 4) for 2023. Statistical significance was determined by one-way ANOVA (*p* < 0.05). Asterisks indicate significant differences among treatments: * denotes significant difference between the control and grass cover treatments, ** denotes significant difference between the control and the other treatments, depending on season and sampling date.

Leaf area index (LAI) was used to characterize seasonal changes in grapevine vegetative growth under the different vineyard floor management treatments. The seasonal dynamics of the LAI were monitored during the 2022–2024 growing seasons under different weed management treatments (Figure 1).

In all seasons, LAI increased from budburst toward fruit set, followed by temporal fluctuations associated with canopy management practices. In 2022, the LAI increased sharply until fruit set, reaching maximum values of 1.34 m^2^ m^−2^ in the control treatment (A) and 1.08 m^2^ m^−2^ in the full cover tall fescue treatment (D). A subsequent decline was observed in Stage 1 following shoot positioning and hedging, after which the LAI increased again but at Stage 2, slightly decreased following leaf removal. The LAI values in the control remained higher than in the tall fescue treatments throughout the season, with a significant difference between the control (A) and treatments C and D as detected in July 2022.

Overall, LAI was lower in 2023 than in 2022 across all the treatments. Within the 2023 season, it increased gradually from the early measurements and reached its highest values at Stage 2. Differences between the control and the selective herbicide treatments appeared on several sampling dates, especially after canopy management operations. The full-cover tall fescue treatment (D) generally produced the lowest LAI during the season.

The seasonal pattern in 2024 was similar, with LAI rising moderately during Stage 1 and more clearly during Stage 3. Across most measurements, the control maintained higher LAI than the selective herbicide and tall fescue treatments. Significant differences among treatments appeared at several time points, particularly during Stage 3. Nevertheless, LAI remained below the values observed in 2022 across all treatments. Overall, the differences in LAI between the control and the perennial turfgrass treatments became progressively greater over the three experimental seasons, indicating a cumulative effect of long-term vineyard floor management on vine vegetative growth.

#### LAI Linear Mixed-Effect Analysis

The results presented in Table 1 show that LAI was significantly affected by season (F_2,1281_ = 243.37, *p* < 0.001), treatment (F_3,12_ = 9.75, *p* = 0.002), and DOY (F_4,1281_ = 162.87, *p* < 0.001), whereas the effect of blocks was not significant (F_4,12_ = 0.94, *p* = 0.472). The significant season × DOY interaction (F_8,1281_ = 57.04, *p* < 0.001) indicated that the seasonal LAI curves differed among seasons. In addition, the treatment × season interaction was significant (F_6,1281_ = 8.89, *p* < 0.001), indicating that the effect of treatment on LAI differed among seasons.

When averaged across seasons, estimated marginal mean LAI was 1.056, 0.88, 0.783, and 0.759 for treatments A, B, C, and D, respectively (Table 2). Treatment A had significantly higher LAI than treatments B, C, and D (Tukey-adjusted *p* ≤ 0.004), whereas no significant differences were detected among treatments B, C, and D (*p* ≥ 0.08). However, because of the significant treatment × season interaction, these overall treatment means should be interpreted as averages across season-specific treatment responses.

Model-estimated seasonal mean LAI, calculated over the DOY range common to all seasons, decreased from 1.066 in 2022 to 0.800 in 2023 and 0.706 in 2024. All pairwise differences among seasons were significant (Tukey-adjusted *p* < 0.001).

### 2.2. Stem Water Potential (Ψstem)

**Figure 2:**
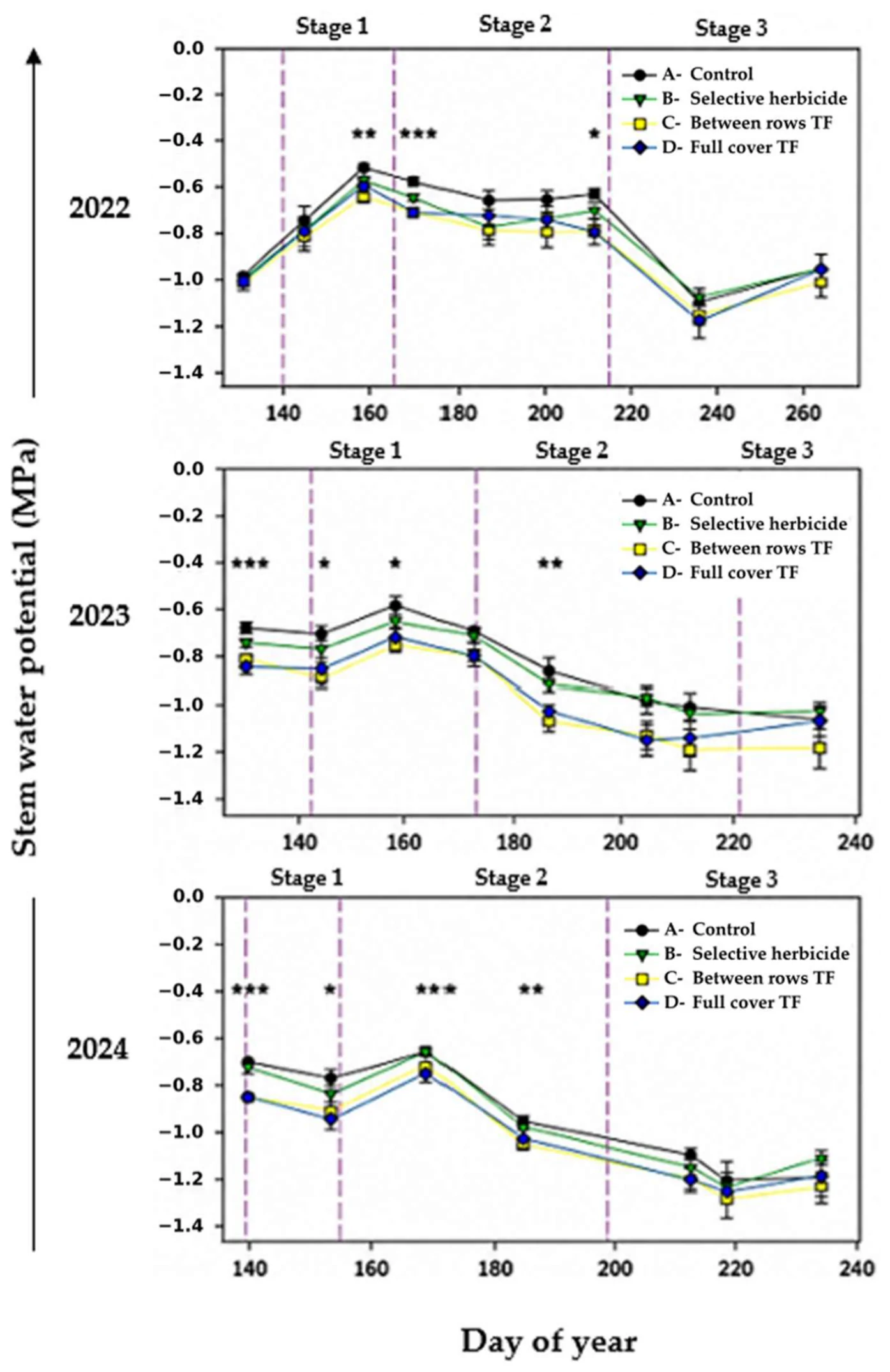
Seasonal dynamics of stem water potential (Ψstem) under different treatments during the 2022 (**upper**), 2023 (**middle**), and 2024 (**lower**) growing seasons. Treatments included a control (A), a selective herbicide (B), tall fescue between rows (C) and full cover tall fescue (D). The X-axis indicates the day of the year, and the fruit development stages (Stages 1–3) are shown above each graph. Each data point represents the mean of treatment plots, with each plot value based on measurements from three vines; *n* = 5 plots in 2022 and 2024 and *n* = 4 plots in 2023. Statistical significance was determined by ANOVA (*p* < 0.05). Asterisks indicate significant differences among treatments: * indicates significant differences between the control and perennial turfgrass treatment treatments, ** indicates significant differences between the control (A) and tall fescue between rows (C) treatments, and *** indicates significant differences among the grass cover treatments (C and D) and/or between the control (C) and Selective herbicide (B) treatments, depending on the season and sampling date.

Across the three growing seasons (2022–2024), seasonal stem water potential (Ψstem) followed a similar temporal pattern, becoming progressively more negative as the season advances (Figure 2). In all seasons, the control treatment generally resulted in less negative Ψstem values, as compared to the perennial turfgrass treatments, although statistically significant differences were observed only on specific measurement dates.

At the beginning of the 2022 growing season, the Ψstem was more negative than expected for this stage of the season, likely due to a delay in the application of the first irrigation. During Stage 1, the values increased (became less negative) and began to stabilize, reaching approximately −0.6 MPa by the end of Stage 1, followed by a moderate decline during Stage 2 (Figure 2). Approximately two weeks before harvest, the Ψstem decreased in all treatments, reaching −1.1 MPa. Following harvest, values increased to approximately −1.0 MPa. Throughout the season, the Ψstem values measured in the control treatment were the least negative. Statistically significant differences were observed on the three measurement dates. On day of year (DOY)159, the Ψstem in the control treatment was significantly greater than that measured in treatment C. In the subsequent measurement in June 2022, the Ψstem in the control was significantly greater than that in Selective herbicide treatment and the tall fescue between rows, whereas Full-cover tall fescue treatment resulted in significantly lower values compared with the other treatments. In the measurements conducted close to veraison, the Ψstem in the control treatment was significantly greater than that in the perennial turfgrass treatment (C and D).

The 2023 growing season began with Ψstem values ranging between −0.66 and −0.81 MPa. Despite a transient increase in early June, the season was characterized by a moderate decline toward values close to −1.0 MPa (Figure 2).

The highest Ψstem values throughout the season were recorded in the control treatment (A), which differed significantly from those in the perennial turfgrass treatments (C and D) during the first three measurement dates of the season and in the measurement conducted in July 2023. In this measurement, the selective herbicide treatment (B) also differed from the tall fescue between rows (C). In the first measurement, Selective herbicide differed significantly from Full-cover tall fescue treatment (D). Notably, although the control treatment never differed statistically from Selective herbicide, a clear trend could be identified. Until mid-Stage 2, Selective herbicide resulted in lower Ψstem values than the control did, whereas by the end of Stage 2, the values in selective herbicide were higher than or similar to those in the control. The growing season of 2024 was also characterized by a moderate decline in Ψstem throughout the season, except for a temporary increase in mid-June (Figure 2). In the first four measurements of the season, the control treatment differed significantly from the grass-covered treatments. In the first and third measurements, Selective herbicide treatment also differed significantly from the perennial turfgrass treatment.

As in the 2023 season, the selective herbicide (B) treatment initially produced more negative Ψstem values than the control. However, later the Ψstem converged and, in the final measurement, even exceeded it. Across both 2023 and 2024, the selective herbicide treatment tended to show an intermediate seasonal pattern. Ψstem was initially more negative than the control and gradually approached the values measured in the control toward the end of the growing season.

#### Ψstem Linear Mixed-Effect Analysis

The results presented in Table 3 show that Ψstem was significantly affected by season (F_2,1233_ = 165.47, *p* < 0.001), treatment (F_3,55_ = 6.26, *p* = 0.008), and DOY (F_4_,_1233_ = 358.47, *p* < 0.001), whereas the effect of block was not significant (F_4,55_ = 0.43, *p* = 0.782). The significant season × DOY interaction (F_8,1233_ = 50.40, *p* < 0.001) indicated that the seasonal Ψstem curves differed among seasons. In contrast, the treatment × season interaction was not significant (F_6,1233_ = 1.52, *p* = 0.167), indicating no evidence that treatment effects differed among seasons.

The findings presented in Table 4 indicate that across seasons, estimated marginal mean Ψstem was −0.759, −0.789, −0.873, and −0.855 for treatments A, B, C, and D, respectively. Treatments A and B did not differ significantly from each other (*p* = 0.335), nor did treatments C and D (*p* = 0.721). In contrast, both A and B differed significantly from C and D (Tukey-adjusted *p* ≤ 0.002).

Model-estimated seasonal mean Ψstem, calculated over the DOY range common to all seasons, shifted from −0.755 in 2022 to −0.871 in 2023 and −0.953 in 2024. All pairwise differences among seasons were significant (Tukey-adjusted *p* < 0.001).

### 2.3. Yield and Its Components

At the 2022 harvest, the highest yield was recorded in the control treatment, although the differences among treatments were not statistically significant (Table 5). No differences were observed in the number of clusters per vine or in cluster weight. However, compared with those in the perennial turfgrass treatments (C and D), berry weight in the control treatment (A) was significantly higher. No differences were detected in total soluble solids among treatments, whereas must pH was significantly lower in the full-cover tall fescue treatment (D) than in the control (A).

During the 2023 season, yield decreased compared with 2022, a trend observed throughout the country (Table 6). The yield reduction was accompanied by lower LAI values than in 2022, as described above (Figure 1). Yield was significantly lower in both tall fescue treatments (C and D) than in the control (A), whereas the selective herbicide treatment (B) showed an intermediate response and did not differ significantly from the control (A). The number of clusters per vine was greater in the control treatment (A), although the differences were not statistically significant. The average cluster weight was highest in the control treatment (A) and lowest in the Full-cover tall fescue treatment. With respect to most quality parameters, no statistically significant differences were observed among treatments.

During the 2024 season, yield declined markedly across all treatments (Table 7). The preceding winter and growing season were characterized by distinct rainfall and chilling conditions, with cumulative rainfall of 434.9–503.2 mm and chilling accumulation of 76-144 CU across the two meteorological datasets (Supplementary Table S1; Figures S1–S4). These seasonal conditions provide important environmental context for the overall decline in productivity; however, the greater reduction observed in the perennial tall fescue treatments (C and D) indicates an additional treatment-associated effect. However, the reduction was more pronounced in the perennial turfgrass treatments, which differed significantly from the control (A). The number of clusters per vine in the control treatment (A) was nearly double that in the perennial tall fescue treatments (C and D) and differed significantly from that in the other treatments. The cluster weight in the control was significantly greater than that in treatment D, and the number of berries per cluster was also greatest in the control (A), differing significantly from that in treatment C. No statistically significant differences were observed in the total sugar content; however, compared with those in 2022, the grapes in the control treatment were significantly less acidic.

### 2.4. Correlations Among Yield Components

Correlation analysis was performed to identify the yield component primarily responsible for the yield differences among treatments across the three growing seasons. In the 2022 season, a strong positive correlation was found between the number of clusters per vine and yield (r = 0.95; Table 8).

In addition, positive correlations were observed between cluster weight and yield (r = 0.77), number of clusters per vine (r = 0.53), and number of berries per cluster (r = 0.70). Correlations involving the number of berries per cluster were statistically significant and indicated positive relationships with yield (r = 0.84), number of clusters per vine (r = 0.70), and cluster weight (r = 0.84), as well as a negative relationship with berries (r = −0.48). During the 2023 season, the number of clusters strongly correlated with yield (r = 0.96). Positive correlations were also observed between cluster weight (r = 0.69) and berry weight (r = 0.67; Table 9). Similarly, yield was positively correlated with cluster weight (r = 0.84) and berry weight (r = 0.63). A moderate positive correlation was found between cluster weight and berry weight (r = 0.53). In contrast, the number of berries per cluster was negatively correlated with berry weight (r = −0.69).

During the 2024 season, a strong positive correlation was also observed between yield and the number of clusters (r = 0.98) as well as cluster weight (r = 0.86; Table 10).

In addition, positive correlations were detected between the number of berries per cluster and yield (r = 0.79), number of clusters (r = 0.77), and cluster weight (r = 0.85). A positive correlation was observed between the number of clusters and cluster weight (r = 0.78), as well as between the number of berries per cluster and berry weight (r = 0.54). Across all three growing seasons, yield was consistently and most strongly associated with the number of clusters per vine (r = 0.95–0.98), whereas the relationships with cluster weight and berry weight were considerably weaker. These results indicate that differences in yield among treatments were driven primarily by changes in cluster number rather than by changes in berry size or cluster weight.

The cumulative effect of the different vineyard floor management strategies became more evident when the relative decline in yield was compared across the three growing seasons (Table 11).

The control treatment (A) showed the smallest cumulative yield reduction (54.9%), whereas the largest decline occurred in the tall fescue between-row (C) treatment (77.2%), followed by the full-cover tall fescue (D) treatment (73.6%). The selective herbicide treatment (B) showed an intermediate cumulative decline of 65.7%.

### 2.5. Relationship Between Seasonal ΨStem and Yield Components

**Figure 3:**
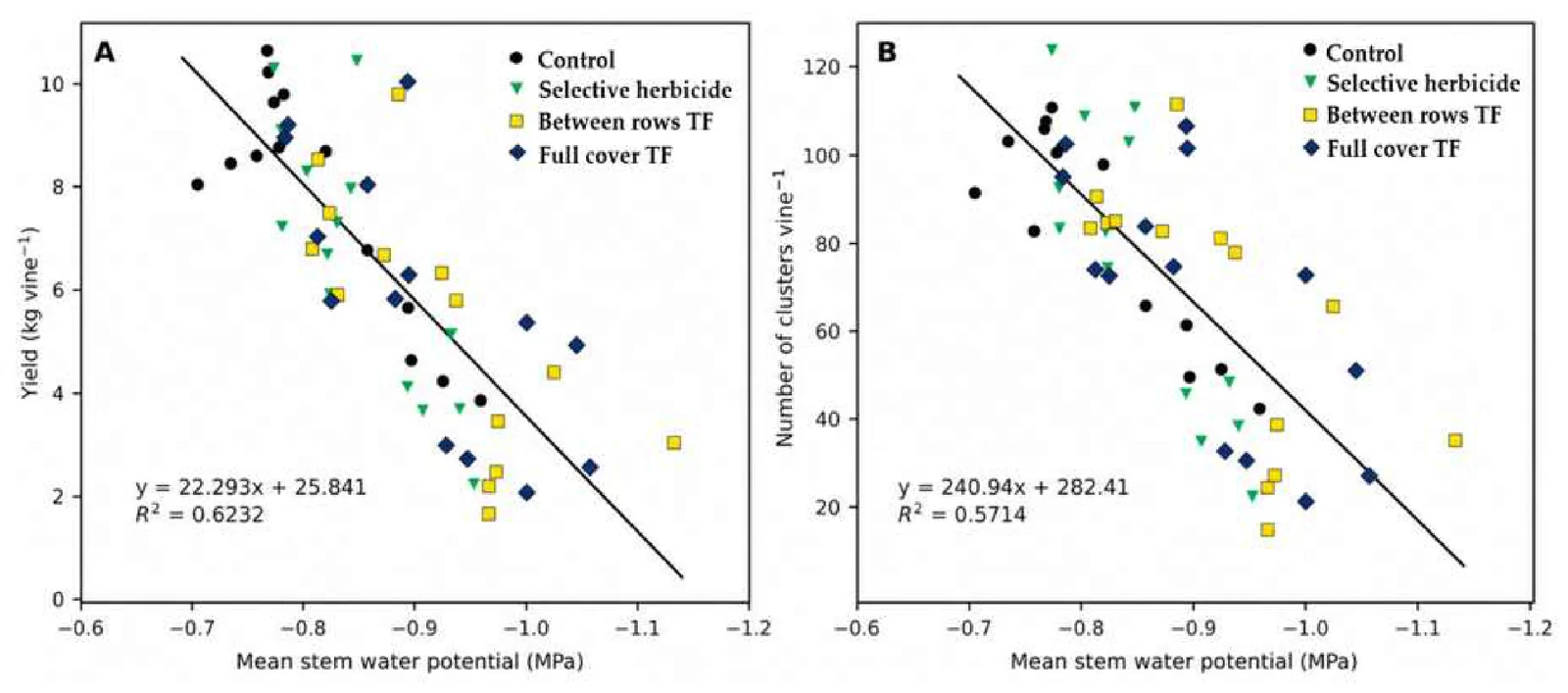
Relationships between seasonal mean stem water potential (Ψstem) and (**A**) yield per vine and (**B**) number of clusters per vine across all treatments and growing seasons (2022–2024). Each point represents one treatment replicate. Solid lines indicate linear regression fits.

Seasonal mean Ψstem was significantly associated with both yield per vine and the number of clusters per vine across all treatments and growing seasons (Figure 3).

Less negative Ψstem values were associated with greater yield and a higher number of clusters per vine. These relationships complement the correlation analysis (Table 8, Table 9 and Table 10), indicating that seasonal yield differences were primarily associated with changes in cluster number.

### 2.6. Wine Color

Wine color is presented only for the third experimental season (2024), when vine performance was considered more physiologically balanced and representative of the long-term influence of the treatments. In addition, during the preceding seasons, the nano-vinification procedure was still being implemented and refined in our laboratory. By 2024, the protocol had been fully standardized, providing the most reliable and methodologically consistent basis for treatment comparison.

Wine color measured during the 2024 season is presented in Table 12. Significant differences among treatments were observed for absorbance at 420, 520, and 620 nm, as well as for total color intensity, whereas hue was not significantly affected by treatment. The highest absorbance values at all measured wavelengths were recorded in the full-cover tall fescue treatment (D), which also exhibited the greatest color intensity, significantly greater than that of the control treatment. Intermediate values were observed in the selective herbicide treatment (B) and the tall fescue between-row treatment (C). Overall, color intensity increased with increasing ground cover intensity.

## 3. Discussion

The present study demonstrates that the long-term response of grapevines to vineyard floor vegetation depends not only on the presence of ground vegetation but also on the duration of competition between the grapevine and the vegetation. While perennial turfgrass remained active throughout the growing season and imposed continuous competition for water, the naturally occurring annual grass promoted by selective herbicide management completed their life cycle before the onset of the dry Mediterranean summer, leaving a protective straw mulch while substantially reducing competition during the period of greatest vine water demand. Consequently, the selective herbicide treatment generally produced intermediate responses in vine water status, canopy development, yield, and wine color attributes. (Figure 1 and Figure 2; Table 8, Table 9, Table 10, Table 11 and Table 12), suggesting that vegetation phenology is a key determinant of vineyard performance under Mediterranean conditions. The consistently more negative Ψstem under the perennial tall fescue treatments (Figure 2), along with corresponding reductions in canopy development and yield, provides strong physiological evidence that competition for available soil water was likely the principal mechanism driving vine responses. Reduced vine water status was accompanied by a lower leaf area index (Figure 1), suggesting that prolonged competition may have contributed to restricted vegetative growth. These findings agree with previous studies reporting that vineyard floor vegetation reduces vine water availability under Mediterranean conditions, and with studies demonstrating that seasonal Ψstem integrates the combined effects of meteorological conditions and vineyard water availability and provides a robust indicator of vine productivity [22,23].

Although the observed responses are consistent with greater competition for available water under perennial tall fescue, other interspecific interactions cannot be excluded. Vineyard cover crops may interact with grapevines through competition for water, nutrients, and rooting space, as well as through changes in soil microbial processes and, potentially, allelopathy [24]. Tall fescue has shown competitive effects on grapevine physiology and productivity, although distinguishing resource competition from other plant–plant interactions under field conditions remains difficult [25]. More broadly, evidence for allelopathic effects of cover crops under field conditions remains limited compared with laboratory studies [26]. Because the present study did not measure allelochemicals, root interactions, nutrient uptake, or cover-crop biomass, allelopathy should therefore be considered a possible additional mechanism rather than a demonstrated explanation for the treatment responses.

The cumulative consequences of these physiological responses became evident in vine productivity. Yield declined in all treatments during the three-year experiment, with the smallest cumulative reduction occurring in the control and larger reductions in both perennial turfgrass treatments; among them, the tall fescue between-row treatment showed the greatest decline (Table 11). The lower productivity observed across treatments in 2024 corelated with distinct seasonal rainfall, temperature, and chilling conditions (Supplementary Figures S1–S4). Therefore, part of the year-to-year variation in yield may reflect common climatic effects. However, the substantially greater reduction observed under perennial turfgrass indicates that prolonged competition for water amplified the impact of these unfavorable environmental conditions. Correlation analysis (Table 8, Table 9 and Table 10) consistently identified cluster number as the yield component most strongly associated with total yield, while seasonal Ψstem was closely related to both yield and cluster number (Figure 3). These findings are consistent with Ohana-Levi et al. [27], who demonstrated that seasonal yield responses are primarily mediated through changes in cluster number, and with previous studies showing that vine water deficits reduce bud fruitfulness and inflorescence initiation, thereby affecting cluster number in the following season [28,29].

Improved wine color intensity in the perennial turfgrass treatments (Table 8) illustrate the well-established trade-off between grape yield and fruit quality under moderate water limitation. Lower vine vigor and a more open canopy enhance light penetration into the fruiting zone and promote the accumulation of anthocyanins and other phenolic compounds [23,30,31]. However, the improvement in wine color observed under perennial turfgrass was accompanied by substantial yield penalties, particularly after three consecutive seasons of competition. In contrast, the selective herbicide treatment generally maintained intermediate responses (Table 8, Table 9, Table 10, Table 11 and Table 12), suggesting that restricting vegetation competition to winter and spring can preserve part of the quality benefits associated with vineyard floor vegetation while minimizing long-term reductions in productivity.

Several limitations should be considered when interpreting these results. The experiment was conducted over three consecutive seasons in a single commercial vineyard using one cultivar–rootstock combination, one irrigation system, and one set of semiarid Mediterranean conditions. Therefore, the magnitude of the observed responses may differ under other cultivars, soils, climates, or management systems. In addition, soil-water content, vegetation water use, root distribution, nutrient uptake, ground-cover biomass, and allelochemical activity were not measured directly, so the observed physiological responses cannot be attributed exclusively to a single below-ground mechanism. Broader ecosystem-service effects, including changes in soil organic matter, erosion, infiltration, biodiversity, and microbial activity, were also not quantified in this study. Future work integrating soil-water dynamics, root interactions, vegetation biomass, nutrient competition, and species-specific biochemical effects would help clarify the relative contribution of these mechanisms and improve the generalization of the findings.

From a practical perspective, these findings suggest considering vineyard floor vegetation management alongside irrigation management rather than independently. The observed differences in vine water status indicate that active vineyard floor vegetation may increase competition for available soil water. Integrating meteorological conditions, canopy development, and vine water status into irrigation decision-making may therefore help account for this additional competitive pressure. Future research should directly quantify the seasonal water use of different vineyard floor vegetation types and determine whether irrigation adjustments can offset this competition while maintaining vineyard productivity. Ultimately, irrigation recommendations should consider the water requirements of the entire vineyard ecosystem, including both the grapevine and vineyard floor vegetation, rather than those of the grapevine alone [23,31]. Selective herbicide application conserved resident winter vegetation while moderately affecting vine water status, canopy development, and overall productivity. Previous studies [11,12] have shown that resident vineyard vegetation can also increase soil organic matter, protect soil, and support various ecosystem functions. However, the current study did not measure these potential benefits and should assess them directly in future research.

Overall, the three-year results indicate that perennial tall fescue was associated with stronger reductions in vine water status (more negative values), lower canopy development, and productivity than the selective herbicide treatment. These responses are consistent with prolonged resource competition, particularly for water, although contributions from nutrient competition, root interactions, or other interspecific mechanisms cannot be excluded. The magnitude of these responses should therefore be validated across additional cultivars, soils, irrigation regimes, and Mediterranean environments. Conclusively, in this study, over three consecutive growing seasons (i.e., in 2022, 2023, and 2024), the effects of vineyard floor management alteration on grapevine performance were analyzed, primarily through competition for water under Mediterranean conditions. The results showed that tall fescue maintained during the growing season progressively reduced leaf area index and Ψstem and was associated with the largest cumulative yield losses. The greatest decline occurred in the tall fescue between-row treatment, followed by the full-cover treatment. Across seasons, yield was most closely associated with cluster number, and both variables (yield and cluster number) declined as seasonal Ψstem became more negative. Although the full-cover tall fescue treatment resulted in higher wine color intensity in the final season, this improvement was observed with substantial loss of productivity. Therefore, wine color intensity did not provide a balanced outcome under the conditions of this study. Selective herbicide management produced a more moderate response that favored resident annual winter grasses that senesced before the dry summer. Vine water status, canopy development, yield, and wine color generally remained between those of the sprayed control and the perennial grass treatments. These findings suggest that the seasonal duration of active ground cover is an important factor influencing grapevine response. Under semiarid Mediterranean conditions, limiting active ground cover to the wettest part of the year may reduce prolonged competition with grapevines compared with maintaining perennial turf through summer. This conclusion should be tested across cultivars, soils, and irrigation regimes. Future studies should directly quantify vegetation water use, soil water dynamics, ground-cover biomass, and soil-related ecosystem services before broader mechanistic, or management conclusions are made.

## 4. Materials and Methods

### 4.1. Experimental Design and Treatments

**Figure 4:**
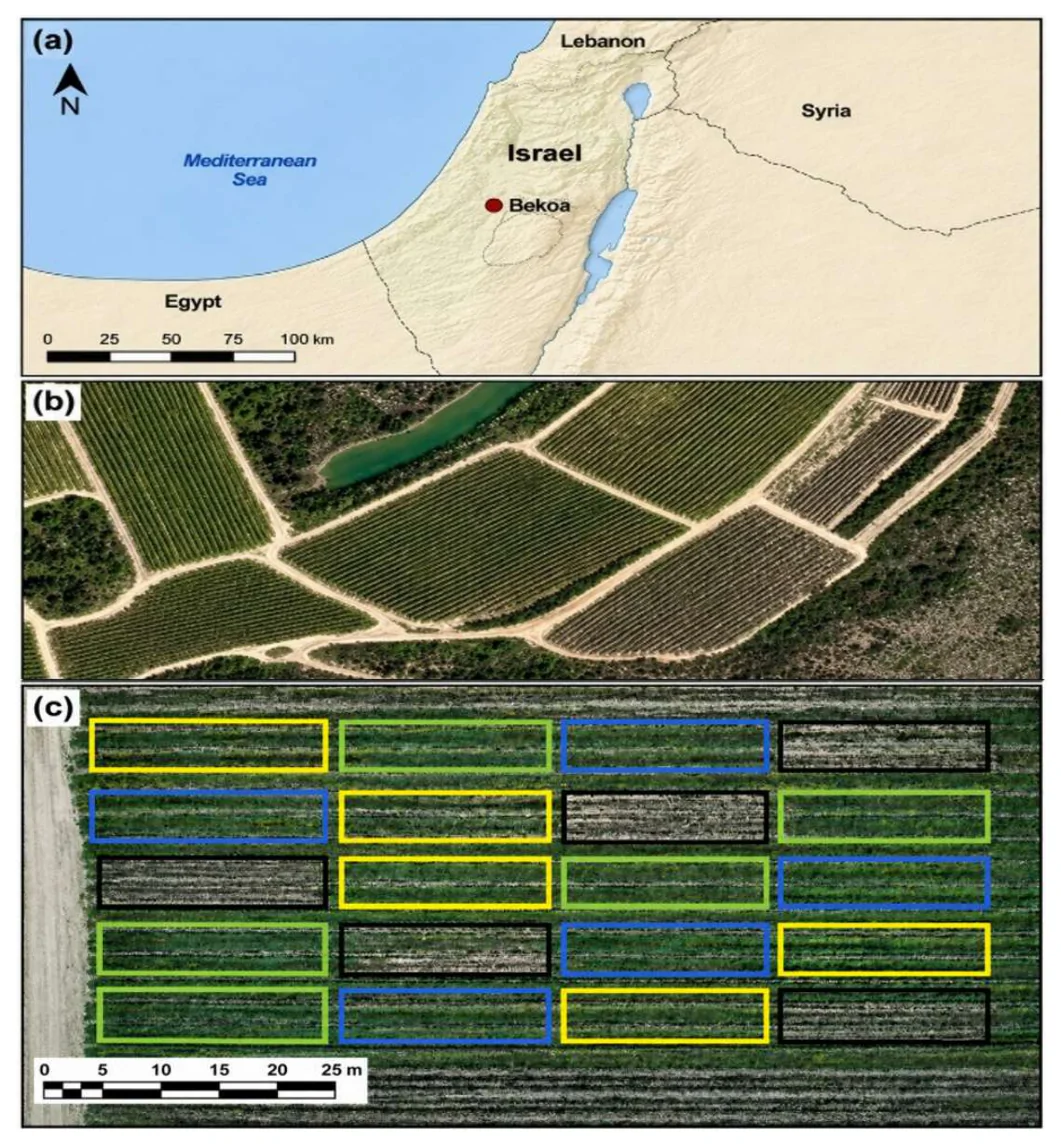
(**a**) Map of Israel (**b**) Location of the experimental plot in Bekoa, and (**c**) Aerial drone image of the experimental vineyard plot in Bekoa. Black rectangles indicate sprayed control plots, green rectangles indicate selective herbicide treatment, yellow rectangles indicate tall fescue between rows, and blue rectangles indicate full ground cover with tall fescue.

The field trial was located in a commercial *Vitis vinifera* cv. Shiraz vineyard near Bekoa (Figure 4) in Israel’s central coastal plain (31.82° N, 34.93° E; elevation 156 m a.s.l.). Vines were grafted on 140 Richter rootstock, planted in 2011 at a spacing of 1.5 m × 3 m, and trained as bilateral cordons on a vertical shoot positioning (VSP) trellis. Drip irrigation is delivered via a subsurface line positioned midway between vine rows, 20–30 cm below ground level, with emitters spaced every 75 cm (1.6 L h^−1^). For all treatments, standard cultural practices were uniformly applied, including irrigation, fertilization, and pest and disease management.

#### 4.1.1. Experimental Design

A randomized complete block design was established with four treatments and five blocks (Figure 4). Each treatment plot comprised three adjacent vine rows with 12 vines per row and was considered the experimental unit. Measurements were taken from the designated measurement row, with 10 vines permanently labelled for repeated observations and three vines per plot selected for physiological and vegetative measurements. Yield and cluster number were recorded from 12 vines per plot. Individual vines were treated as subsamples rather than independent replicates. Five plots per treatment were available in 2022 and 2024, whereas four plots per treatment were available for the 2023 harvest analysis. Meteorological conditions for the 2024 experimental season are included in the Supplementary File (Figures S1–S4 and Table S1), including daily rainfall, daily mean temperature, cumulative seasonal rainfall, and chilling accumulation according to the Tzur-Niron Model.

#### 4.1.2. Treatments

- Control (standard herbicide management): Preemergence application of diuron (Diurex, Adama-Agan, Israel, 2.4 kg ai ha^−1^) + oxyfluorfen (Galigan, Adama-Agan, Israel, 0.72 kg ai ha^−1^) was carried out in autumn (≈50 cm on each side of the vine row). Spontaneous weed vegetation remained untouched between rows, until controlled using postemergence herbicides, namely Glufosinate-ammonium (Basta, BASF, Germany, 0.6 kg ai ha^−1^) + oxyfluorfen 0.12 kg ai ha^−1^ during early spring. This was done in order to allow comparisons between bare soil and vegetation treatments.
- Selective herbicide treatment: The broadleaf herbicide carfentrazone-ethyl (Spot-light, FMC, USA, 0.6 kg ai ha^−1^) was applied between rows to selectively control the broad-leaved weeds and allow naturally occurring annual winter grasses to dominate the vegetation, primarily *Avena sterilis*, *Lolium rigidum*, and *Bromus madritensis* while maintaining in-row weed control similar to that of the control treatment. These annual species naturally completed their life cycle before the onset of the dry summer period, leaving a layer of dry straw mulch on the soil surface during summer.
- Tall fescue between rows: Seeding of *Festuca arundinacea* ‘Avenger II’ (Mountain View Seeds Ltd., Salem, OR, USA) between vine rows was conducted at 2.5 kg ha^−1^. Seeding was performed manually in late November using a handheld spreader, followed by light incorporation with forks to enhance seed–soil contact. A second seeding in December improved ground coverage. In-row herbicide management was identical to that of the control.
- Tall fescue full cover: Seeding of *F. arundinacea* both between and within vine rows was carried out as described above, without any herbicide use. Vegetation management relied exclusively on mowing.

**Figure 5:**
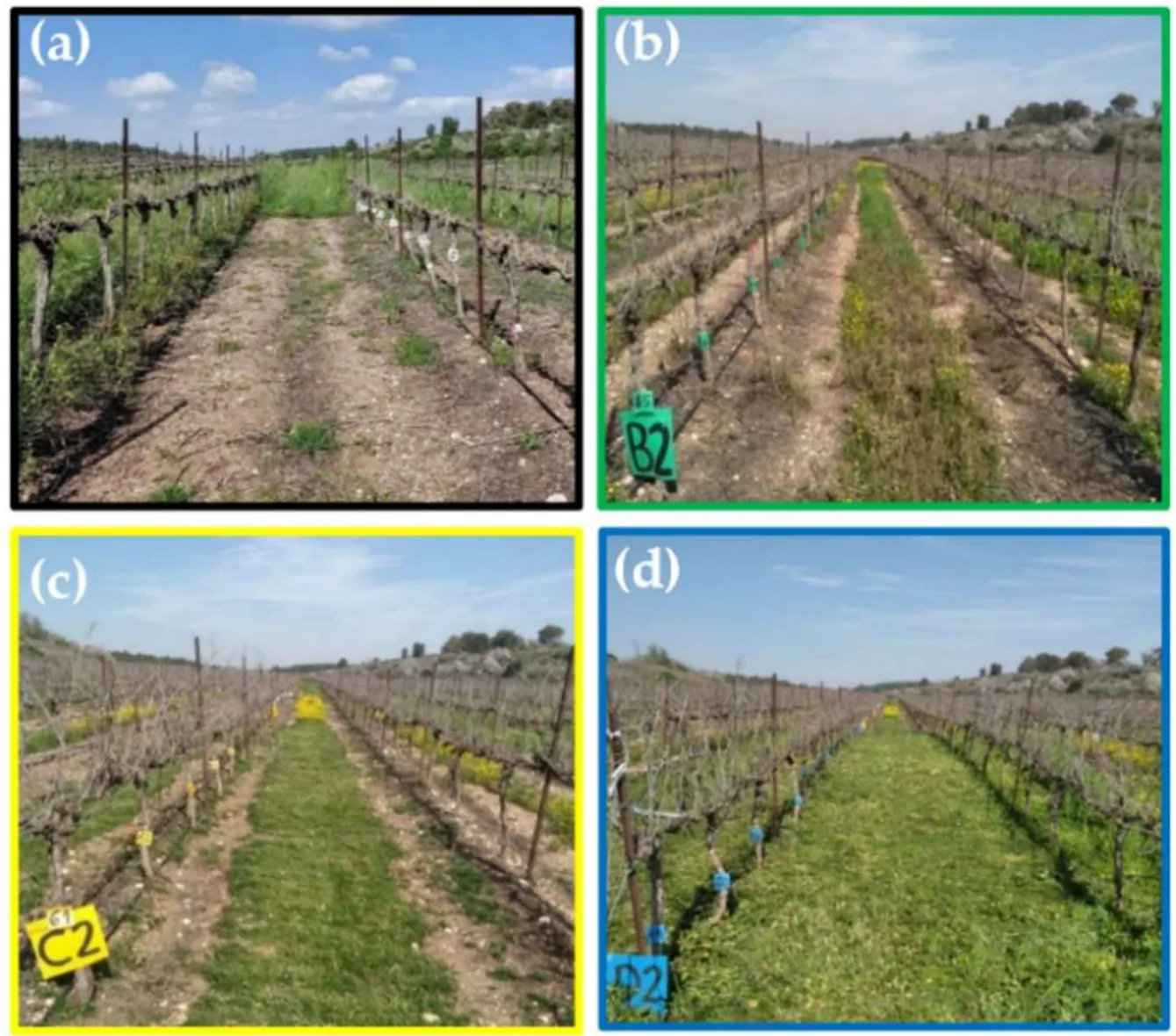
The different experimental treatments: Each rectangle delineates the boundaries of a replicate. The treatments are marked by color: (**a**) black: control, (**b**) green: selective herbicide (encouraged grasses), (**c**) yellow: tall fescue (*Festuca arundinacea*) between rows, and (**d**) blue: tall fescue sown as full ground cover.

Each rectangle delineates the boundaries of a replicate. Treatments were marked by color: Black-Control (a), green-selective herbicide (b), yellow-tall fescue between rows (c), blue-tall fescue full cover (d) (Figure 5).

### 4.2. Vine Vegetative and Physiological Measurements

#### 4.2.1. Leaf Area Index (LAI)

From May to September, LAI was measured biweekly on three vines per treatment plot, selected from the designated measurement row, using a SunScan canopy analyzer (Delta-T Devices Ltd., Burwell, Cambridge, UK) The LAI was calculated as the ratio of the leaf surface area to ground area (m^2^ m^−2^), adjusted for row spacing and unshaded gaps [32].

#### 4.2.2. Stem Water Potential (ΨStem)

Ψstem was measured biweekly from May to September on the same three vines per treatment plot used for physiological measurements, using a pressure (Model 600, PMS Instrument Company, Albany, OR, USA; MRC 3000, MRC Ltd., Holon, Israel). Mature, sun-exposed leaves were enclosed in plastic bags covered with aluminum for at least 2 h prior to measurement to allow equilibration of the leaf and Ψstem.

#### 4.2.3. Linear Mixed-Effect Analysis

Effects on LAI and Ψstem were analyzed separately using a linear mixed-effects model [33,34] to account for the hierarchical structure of the experiment and repeated measurements of individual trees. The Block × Treatment combination was considered the experimental unit, with individual trees treated as subsamples within each experimental unit. Block, season, treatment, and their interaction (treatment × season) were included as fixed effects, while random intercepts were included for the Block × Treatment experimental unit and for individual tree nested within the experimental unit. Since LAI and Ψstem are seasonally dynamic, their seasonal trajectories were modeled as a nonlinear function of day of year (DOY) using a natural cubic spline with four degrees of freedom, and a season × DOY interaction was included to allow the seasonal trajectory to differ among seasons (following a similar approach to Welham et al. [35](. Model parameters were estimated using restricted maximum likelihood (REML), and the significance of fixed effects was assessed using sequential F-tests. Post-hoc comparisons among treatments were performed using estimated marginal means with Tukey adjustment for multiple comparisons. Because the seasonal trajectory differed among seasons, comparisons among seasons were based on model-estimated seasonal means calculated over the DOY range common to all seasons, with each day within this range weighted equally; pairwise differences among seasons were subsequently evaluated using Tukey-adjusted comparisons. Statistical analyses were conducted in R version 4.4.3 using the nlme [36,37] and emmeans [38] packages.

### 4.3. Yield and Fruit Composition

At harvest, the total yield and number of clusters were recorded separately for each experimental vine. Measurements were obtained from 240 vines per season (12 measurement vines × 5 replicates × 4 treatments), resulting in a total of 720 vine observations over the during 2022 and 2024 growing seasons. In 2023, 12 measurement vines × 4 replicates × 4 treatments were evaluated, resulting in a total of 192 vine observations, as the fifth replicate was lost due to inadvertent mechanical harvesting. Mean cluster weight was calculated by dividing the total yield per vine by the corresponding number of clusters. In addition, 24 clusters were randomly sampled from each replicate (two clusters per vine). A random subsample of 100 berries was collected from these clusters and weighed to determine the mean berry weight. The mean number of berries per cluster was then estimated by dividing the mean cluster weight by the mean berry weight. The remaining berries were crushed, and the must was analyzed for total soluble solids (Brix) and pH using a pocket refractometer (PAL series, ATAGO Co., Ltd., Tokyo, Japan) and a pH meter (CyberScan pH 500, Eutech Instruments Pte Ltd., Singapore).

### 4.4. Micro-Vinification and Wine Analysis

To assess potential effects on wine color attributes, micro-vinifications were carried out using the micro-vinification system developed at the Eastern Regional R&D Center, Ariel University [39]. From each replicate, 1.6 kg of grapes were fermented in 2 L glass fermenters at 24 °C with *Saccharomyces cerevisiae* (0.25 g kg^−1^). After nine days, the wines were pressed, racked, and bottled (375 mL). Wine color attributes were determined spectrophotometrically (UV–VIS GENESYS 10, Thermo Scientific, Waltham, MA, USA) at 420, 520, and 620 nm to calculate color density (A_420_ + A_520_ + A_620_) and hue (A_420_/A_520_).

### 4.5. Data Analysis

Statistical analyses and standard error calculations were performed on the means derived from all five replicates. Analysis of grapevine parameters, yield and its components was conducted using one-way ANOVA (with α = 0.05 and *p* < 0.05), followed by Tukey’s post hoc test to determine the differences between the means of the various evaluated parameters. Linear regression analysis was additionally performed to examine the relationships between seasonal mean Ψstem and yield components.

### 4.6. Declaration of Generative AI Use

During the preparation of this manuscript, the authors used ChatGPT (OpenAI) for language editing, improving clarity, and assisting with the presentation of text and figures. All statistical analyses were performed and verified by the authors using JMP, version 16.0.0. The authors reviewed and approved the final manuscript and take full responsibility for its content.

## Figures and Tables

**Table 1:** Linear mixed-effects model for leaf area index (LAI).

Effect	F	*p*-Value
Block	0.94	0.472
Season	243.37	<0.001
Treatment	9.75	0.002
DOY	162.87	<0.001
Season × Treatment	8.89	<0.001
Season × DOY	57.04	<0.001

**Table 2:** Estimated marginal means of LAI by treatment and season.

Factor	Level	Estimated Marginal Mean (m^2^ m^−2^) *	Tukey Group **
Treatment	A-Control	1.056	b
	B-Selective herbicide	0.880	a
	C-Tall fescue between rows	0.783	a
	D-Tall fescue full cover	0.759	a
Season	2022	1.066	c
	2023	0.800	b
	2024	0.706	a

* Treatment means are estimated marginal means averaged across blocks and seasons. Seasonal means are model-estimated means averaged across blocks, treatments, and the DOY range common to all seasons, with each day weighted equally. ** Within each factor, levels sharing the same letter were not significantly different according to Tukey-adjusted pairwise comparisons (α = 0.05).

**Table 3:** Linear mixed-effects model for stem water potential (Ψstem).

Effect	F	*p*-Value
Block	0.43	0.782
Season	165.47	<0.001
Treatment	6.26	0.008
DOY	358.47	<0.001
Season × Treatment	1.52	0.167
Season × DOY	50.40	<0.001

**Table 4:** Estimated marginal means of Ψstem by treatment and season.

Factor	Level	Estimated Marginal Mean (MPa) *	Tukey Group **
Treatment	A-Control	−0.759	a
	B-Selective herbicide	−0.789	a
	C-all fescue between rows	−0.873	b
	D-Tall fescue full cover	−0.855	b
Season	2022	−0.755	a
	2023	−0.871	b
	2024	−0.953	c

* Treatment means are estimated marginal means averaged across blocks and seasons. Seasonal means are model-estimated means averaged across blocks, treatments, and the DOY range common to all seasons, with each day weighted equally. ** Within each factor, levels sharing the same letter were not significantly different according to Tukey-adjusted pairwise comparisons (α = 0.05).

**Table 5:** Harvest data and must quality, September 2022 *.

Treatment	pH	Total Soluble Solids (°Bx)	Number of Berries/Cluster	Berry Weight (g)	Cluster Weight (g)	Number of Clusters/Vine	Yield (kg/Vine)
A—Control	3.55 ^a^	20.28	71	1.42 ^a^	101.3	97	9.43
B-Selective herbicide	3.46 ^ab^	19.24	64	1.42 ^ab^	89.8	97	8.72
C-Tall fescue between rows	3.45 ^ab^	19.86	65	1.33 ^b^	86.3	89	7.84
D-Tall fescue full cover	3.41 ^b^	20.20	71	1.33 ^b^	94.3	91	8.63
*p*-Value	0.0015	NS	NS	0.0103	NS	NS	NS

* The data presented in the table represent measurements obtained from 60 vines per treatment, 240 vines in total (5 replicates per treatment, 12 vines per replicate). Different letters indicate statistically significant differences (*p* ≤ 0.05).

**Table 6:** Harvest data and must quality, September 2023 *.

Treatment	pH	Total Soluble Solids (°Bx)	Number of Berries/Cluster	Berry Weight (g)	Cluster Weight (g)	Number of Clusters/Vine	Yield (kg/Vine)
A-Control	3.36	17.73	66	1.35	88.0 ^a^	102	8.86 ^a^
B-Selective herbicide	3.33	17.78	66	1.24	80.2 ^ab^	91	7.35 ^ab^
C-Tall fescue between rows	3.33	17.80	66	1.14	75.0 ^b^	76	5.57 ^c^
D-Tall fescue full cover	3.34	18.40	65	1.17	76.1 ^b^	79	5.80 ^bc^
*p* Value	NS	NS	NS	NS	0.0242	NS	0.0006

* The data presented in the table represent measurements obtained from 48 vines per treatment, 192 vines in total (4 replicates per treatment, 12 vines per replicate). Different letters indicate statistically significant differences (*p* ≤ 0.05).

**Table 7:** Harvest data and must quality, August 2024 *.

Treatment	pH	Total Soluble Solids (°Bx)	Number of Berries/ Cluster	Berry Weight (g)	Cluster Weight (g)	Number of Clusters/ Vine	Yield (kg/Vine)
A-Control	3.59 ^b^	23.76	65.9	1.20	78.7 ^a^	53 ^a^	4.25 ^a^
B-Selective herbicide	3.63 ^ab^	24.26	58.1	1.36	78.4 ^a^	37 ^ab^	2.99 ^ab^
C-Tall fescue between rows	3.66 ^ab^	24.24	51.5	1.24	63.4 ^b^	28 ^b^	1.79 ^b^
D-Tall fescue full cover	3.70 ^a^	24.64	55.2	1.25	68.8 ^ab^	32 ^b^	2.28 ^b^
*p*-Value	0.0283	NS	NS	NS	0.0064	0.0002	0.0088

* The data presented in the table represent measurements obtained from 60 vines per treatment, 240 vines in total (5 replicates per treatment, 12 vines per replicate). Different letters indicate statistically significant differences (*p* ≤ 0.05).

**Table 8:** Correlation matrix describing the relationships among yield components, 2022 season *.

Number of Berries/Clusters	Berry Weight	Cluster Weight	Number of Clusters/Vine	Yield	Component
					Yield
				r = 0.95 *p* < 0.0001	Number of clusters/vine
			r = 0.53 *p* = 0.0286	r = 0.77 *p* = 0.0003	Cluster weight
		r = 0.07 NS	r = −0.44 NS	r = −0.31 NS	Berry weight
	r = −0.48 *p* = 0.05	r = 0.84 *p* < 0.0001	r = 0.70 *p* = 0.0017	r = 0.84 *p* < 0.0001	Number of berries/clusters

* Each cell presents the Pearson correlation coefficient (r) and the *p* value.

**Table 9:** Correlation matrix describing the relationships among yield components, 2023 season *.

Number of Berries/Clusters	Berry Weight	Cluster Weight	Number of Clusters/Vine	Yield	Component
					Yield
				r = 0.96 *p* < 0.0001	Number of clusters/vine
			r = 0.69 *p* = 0.0066	r = 0.84 *p* = 0.0002	Cluster weight
		r = 0.53 *p* = 0.05	r = 0.67 *p* = 0.0084	r = 0.63 *p* = 0.0149	Berry weight
	r = −0.69 *p* = 0.0068	r = 0.25 NS	r = −0.16 NS	r = 0.004 NS	Number of berries/clusters

* Each cell presents the Pearson correlation coefficient (r) and the *p* value.

**Table 10:** Correlation matrix describing the relationships among yield components, 2024 season *.

Number of Berries/Clusters	Berry Weight	Cluster Weight	Number of Clusters/Vine	Yield	Component
					Yield
				r = 0.98 *p* < 0.0001	Number of clusters/vine
			r = 0.78 *p* = 0.0002	r = 0.86 *p* < 0.0001	Cluster weight
		r = −0.05 NS	r = −0.21 NS	r = −0.12 NS	Berry weight
	r = −0.54 *p* = 0.0208	r = 0.85 *p* < 0.0001	r = 0.77 *p* = 0.0002	r = 0.79 *p* < 0.0001	Number of berries/clusters

* Each cell presents the Pearson correlation coefficient (r) and the *p* Value.

**Table 11:** Relative yield decline (%) * across the three growing seasons.

Treatment	Yield (kg Vine^−1^) 2022	Yield (kg Vine^−1^) 2023	Yield (kg Vine^−1^) 2024	Decline 2022 → 2023 (%)	Decline 2023 → 2024 (%)	Overall Decline 2022 → 2024 (%)
A-Control	9.43	8.86	4.25	−6.0	−52.0	−54.9
B-Selective Herbicide	8.72	7.35	2.99	−15.7	−62.3	−65.7
C-Tall fescue between rows	7.84	5.57	1.79	−29.0	−61.0	−77.2
D-Tall fescue full Cover	8.63	5.80	2.28	−32.8	−68.3	−73.6

* Relative decline (%) was calculated from the yield values presented in Table 1, Table 2 and Table 3. Negative values indicate a reduction in yield relative to the previous season or to the 2022 baseline.

**Table 12:** Mean values of wine color attributes for wines produced from Shiraz harvest grapes, September 2024 *.

Treatment	A_420_ (Yellow)	A_520_ (Red)	A_620_ (Blue)	Color Intensity (A_420_ + A_520_ + A_620_)	Hue (A_420_/A_520_)
A-Control	10.32 ^b^	11.32 ^b^	2.73 ^b^	24.4 ^b^	0.91
B-Selective herbicide	11.89 ^ab^	12.30 ^b^	3.15 ^ab^	27.3 ^ab^	1.01
C-Tall fescue between rows	13.67 ^ab^	15.39 ^ab^	3.72 ^ab^	32.8 ^ab^	0.89
D-Tall fescue full cover	15.27 ^a^	16.52 ^a^	4.44 ^a^	36.3 ^a^	0.95
*p*-Value	0.01	0.044	0.009	0.011	NS

* Different letters indicate statistically significant differences (*p* ≤ 0.05).

## Data Availability

The original contributions presented in the study are included in the article; further inquiries can be directed to the corresponding authors.

## Supplementary Materials

The following supporting information can be downloaded at https://www.mdpi.com/article/10.3390/plants15193046/s1, Figure S1: Cumulative chill units at (a) Hulda and (b) MH from 1 November 2023 to 31 March 2024, calculated using the Tzur–Miron model. Seasonal totals were 76 CU at Hulda and 144 CU at MH; Figure S2: Daily rainfall at (a) Hulda and (b) MH from 1 October 2023 to 30 April 2024. Cumulative seasonal rainfall was 503.2 mm at Hulda and 434.9 mm at MH; Figure S3: Daily mean air temperature during 2024 at (a) Hulda and (b) MH. The data show the seasonal transition from cool winter conditions to the warm Mediterranean summer and subsequent autumn–winter cooling; Figure S4: Daily mean air temperature and daily rainfall during 2024 at (a) Hulda and (b) MH. Temperature is shown as a continuous line and rainfall as daily bars, illustrating the concentration of rainfall in the cooler part of the year and the predominantly dry summer period; Table S1: Summary of environmental conditions during the 2023–2024 winter and 2024 growing season at the Hulda and MH meteorological stations.

## Abbreviations

The following abbreviations are used in this manuscript:

CC	Cover Crop
Ai	Active ingredient
TF	Tall Fescue (*Festuca arundinacea*)
LAI	Leaf Area Index
UAV	Unmanned Aerial Vehicle
VSP	Vertical Shoot Positioning
DOY	Day of Year
Ψstem	Stem Water Potential
PAR	Photosynthetically Active Radiation
LVPD	Leaf Vapor Pressure Deficit
