# Supplementary material for: Long-Term Effects of Vineyard Floor Vegetation Management on Grapevine Physiology, Yield and Wine Color Attributes Under Mediterranean Conditions
Source: Plants (Basel). 2026 Oct 5;15(19):3046. doi: 10.3390/plants15193046 (PMC13667049; doi:10.3390/plants15193046)
Supplement: Supplementary file 1 [file plants-15-03046-s001.zip › plants-4528984-supplementary.pdf]

Supplementary Information

Long-Term Effects of Vineyard Floor Vegetation Management on Grapevine Physiology, Yield and Wine Color Attributes Under Mediterranean Conditions

Ronit Yaa'ri [1,2,*], Ido Walk [2,3,*], Moshe Sibony [4], Bat Chen R. Lubin [1,2], Jyoti Solanki [1,2], Yaakov Goldwasser [4], Tovit Rosenzweig [3], Baruch Rubin [4,*] and Yishai Netzer [1,2,*]

[1] Chemical Engineering Department, Ariel University, Ariel 4070000, Israel; ronityar@ariel.ac.il (R.Y.); batchenl@ariel.ac.il (B.C.R.L.); jytsolanky269@gmail.com (J.S.)

[2] Department of Agriculture and Oenology, Eastern R&D Center, Ariel 4070000, Israel; ido.walk@mail.huji.ac.il

[3] Department of Molecular Biology, Faculty of Natural Sciences, Ariel University, Ariel 40700, Israel; tovitro@ariel.ac.il

[4] RH Smith Institute of Plant Sciences & Genetics in Agriculture, Faculty of Agriculture, Food and Environment, The Hebrew University of Jerusalem, Rehovot 7610001, Israel; moshe.sibony@mail.huji.ac.il (M.S.); yaakov.goldwasser@mail.huji.ac.il (Y.G.)

* Correspondence: rubin@mail.huji.ac.il (B.R.); ynetzer@gmail.com (Y.N.);
+Tel.: +972-54-882-0054 (B.R.); +972-54-632-0289 (Y.N.)
† These authors contributed equally to this work.

1. Supplementary environmental information for the 2024 experimental season

We collected data on environmental conditions during the winter season of 2023–2024 and the corresponding calendar year of 2024 from comprehensive meteorological datasets provided by the Hulda and MH weather stations as part of this study. The figures below illustrate key daily metrics, including total daily rainfall, average daily air temperature, and cumulative chilling hours over the specified period. For the chilling accumulation, we used the Tzur–Miron model to accurately calculate values from November 1, 2023, to March 31, 2024. This model helps in understanding the chilling requirements necessary for the successful growth and development of certain plant species. Additionally, total rainfall was recorded between October 1, 2023, and April 30, 2024, providing a comprehensive view of moisture levels over this extended period. It's important to note that the meteorological data from the two stations are presented independently, as each location exhibited differing cumulative values (Table S1.). These variations may reflect the distinct geographical and climatic conditions of each area. This separation facilitates a more nuanced interpretation of the environmental impacts observed in both regions.

Table S1. Summary of environmental conditions during the 2023–2024 winter and 2024 growing season at the Hulda and MH meteorological stations.

Variable	Period	Hulda	MH
Seasonal rainfall	1 Oct 2023–30 Apr 2024	503.2 mm	434.9 mm
Cumulative chilling	1 Nov 2023–31 Mar 2024	76 CU	144 CU
Chilling model	—	Tzur–Miron	Tzur–Miron
Daily mean temperature	2024	Presented in Fig. S3	Presented in Fig. S3
Daily temperature + rainfall	2024	Presented in Fig. S4	Presented in Fig. S4

Note on irrigation. *Seasonal irrigation totals were not included in the environmental files supplied for preparation of this supplement. Therefore, no irrigation amount is reported here. If annual irrigation records are available, they should be added as a separate row in the table above (preferably in mm or $m^3\ ha^{-1}$) and cited in the main manuscript.*

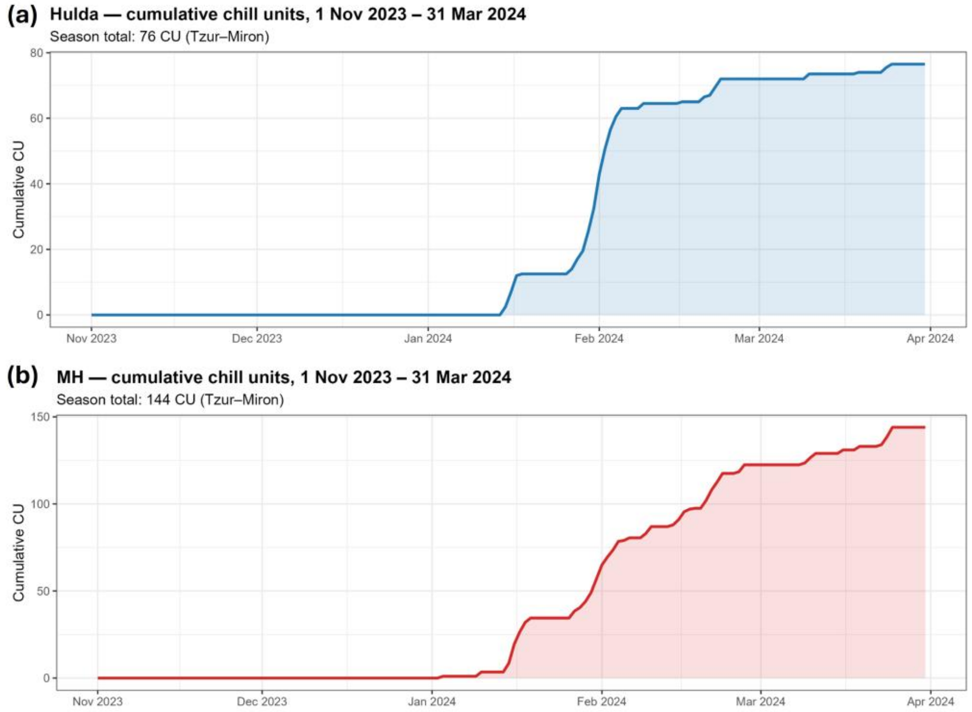

Figure S1. Cumulative chill units at (a) Hulda and (b) MH from 1 November 2023 to 31 March 2024, calculated using the Tzur–Miron model. Seasonal totals were 76 CU at Hulda and 144 CU at MH.

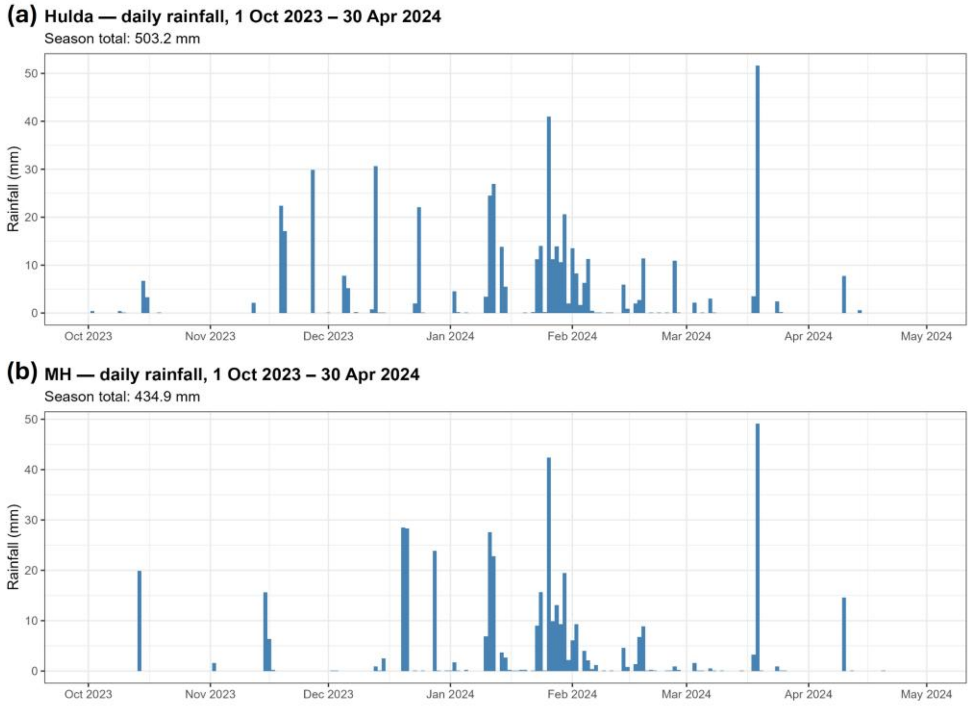

Figure S2. Daily rainfall at (a) Hulda and (b) MH from 1 October 2023 to 30 April 2024. Cumulative seasonal rainfall was 503.2 mm at Hulda and 434.9 mm at MH.

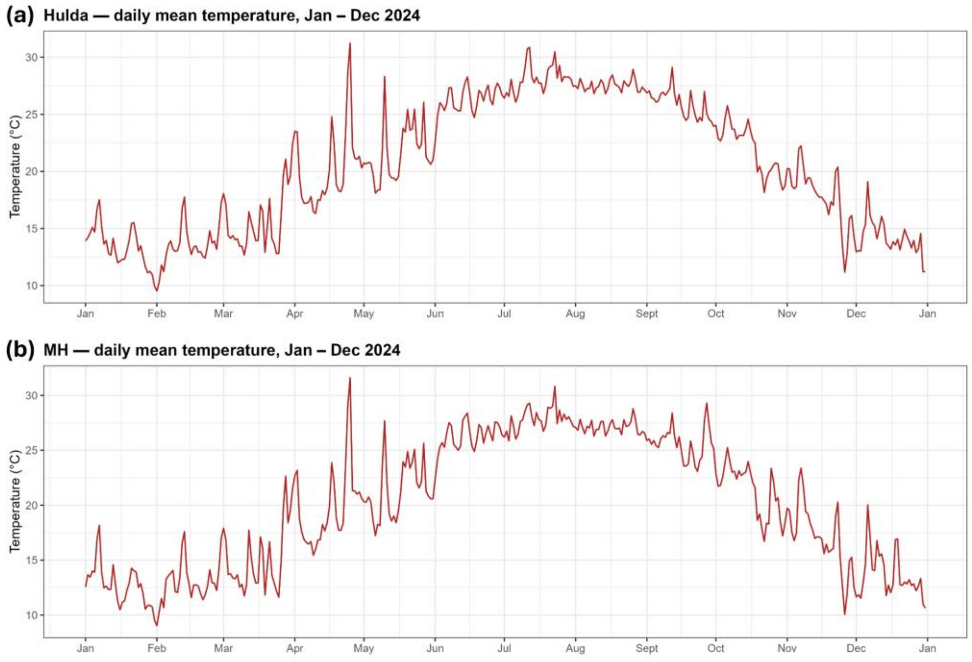

Figure S3. Daily mean air temperature during 2024 at (a) Hulda and (b) MH. The data show the seasonal transition from cool winter conditions to the warm Mediterranean summer and subsequent autumn–winter cooling.

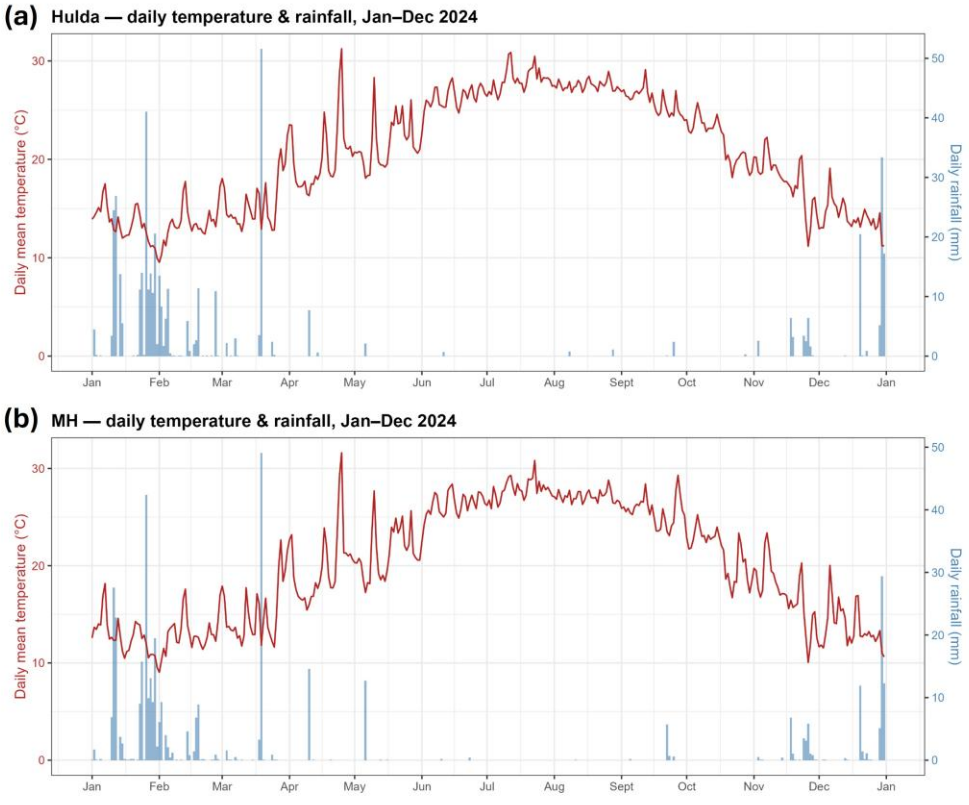

Figure S4. Daily mean air temperature and daily rainfall during 2024 at (a) Hulda and (b) MH. Temperature is shown as a continuous line and rainfall as daily bars, illustrating the concentration of rainfall in the cooler part of the year and the predominantly dry summer period.
